# On the most suitable sites for wind farm development in Nigeria

**DOI:** 10.1016/j.dib.2018.04.144

**Published:** 2018-05-08

**Authors:** T.R. Ayodele, A.S.O. Ogunjuyigbe, O. Odigie, A.A. Jimoh

**Affiliations:** a Power, Energy, Machine, & Drive Research Group, Department of Electrical and Electronic Engineering, Faculty of Technology, University of Ibadan, Ibadan, Nigeria; b Department of Electrical Engineering, Tshwane University of Technology, Private Bag X680 Pretoria 0001, Staatsartillerie Road, Pretoria West, South Africa

## Abstract

The increasing demand for energy and the need for clean and affordable energy in Nigeria have necessitated the need for renewable energy resource assessment and subsequent determination of suitable sites within the country. One of the promising renewable energy resources with good potentials of meeting the energy requirements is wind. One of the main challenges of wind power development in Nigeria is lack of scientific data for policy formulation and decision making that will aid the development of wind power utilization. The data presented in this article were obtained with proper evaluation of the wind resource while taking into consideration environmental, social, and economic factors. The information from the data could be useful for taking optimal site selection decision by the policy makers, government, engineers etc. This will ensure optimal investment and return on investment for wind farm developers.

**Specifications Table**

**Table:** 

**Subject area**	Engineering
**More specific subject area**	Renewable Energy, Multi-criteria decision making, Wind Energy Technology
**Type of data**	Table, charts, maps
**How data was acquired**	Unprocessed secondary data and experts’ survey
**Data format**	Raw, analyzed
**Experimental factors**	The vector maps were converted to raster format which is the acceptable form to ease the evaluation process
**Experimental features**	Wind farm site evaluation with a GIS-based model using interval type-2 fuzzy AHP multi-criteria decision making technique
**Data source location**	Nigerian population commission, Nigeria Meteorological Agency (NIMET) and online databases
**Data accessibility**	This data article contains all the data

**Value of the data**

• Discoveries from the data set could draw the attention of the government to the most suitable states for wind farm establishment for grid integration.
• The data set could serve as a tool for estimating wind energy potential in the country and development of renewable energy map.
• The data set could serve as a reference for the utilization of wind resource for energy generation by investors and energy companies.
• The data and findings could serve as a reference for government policies and planning.
• The data set could be used for educational and instructional purposes.

## 1 Data

**Fig. 1:**
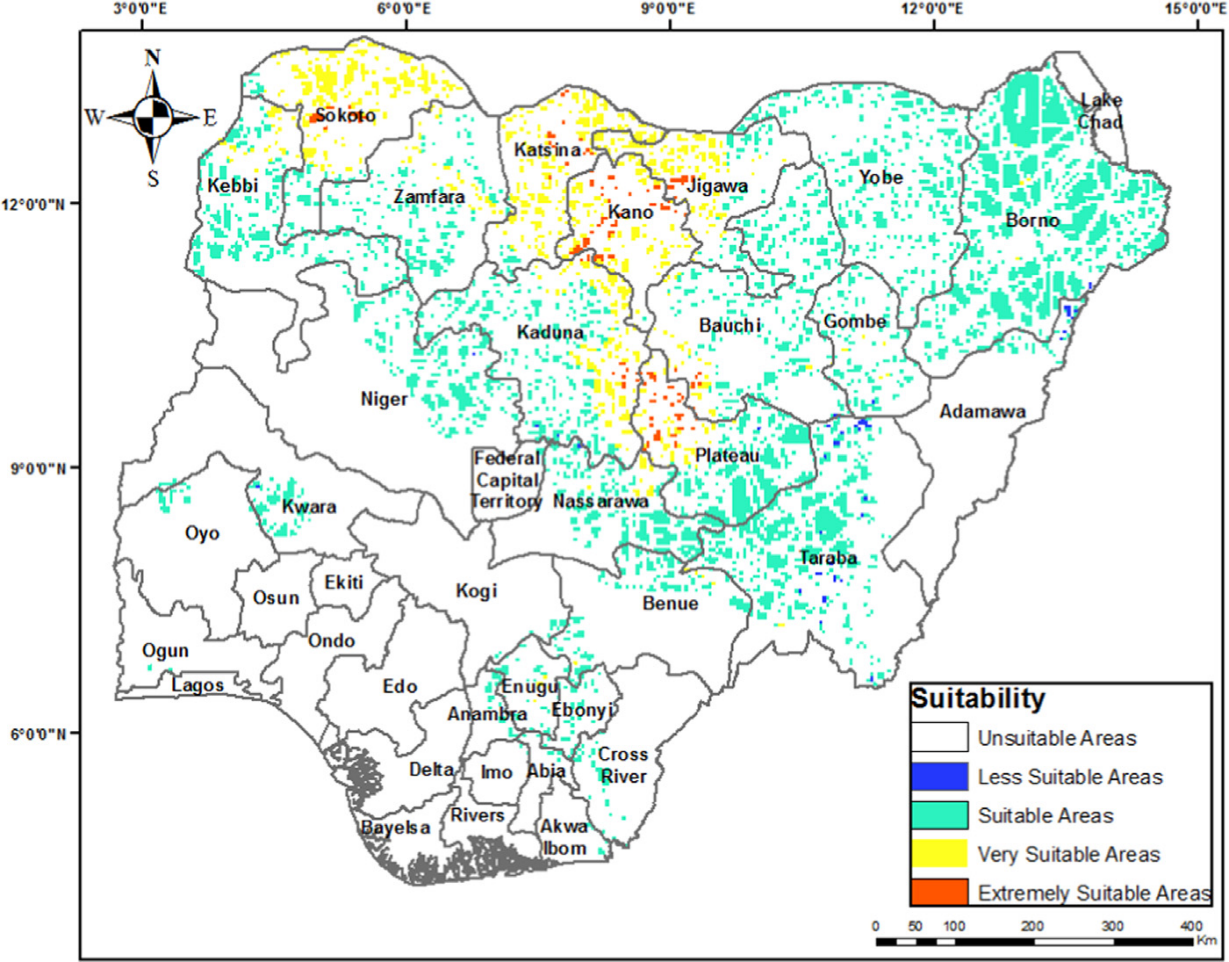
Wind farm suitability map for Nigeria.

**Fig. 2:**
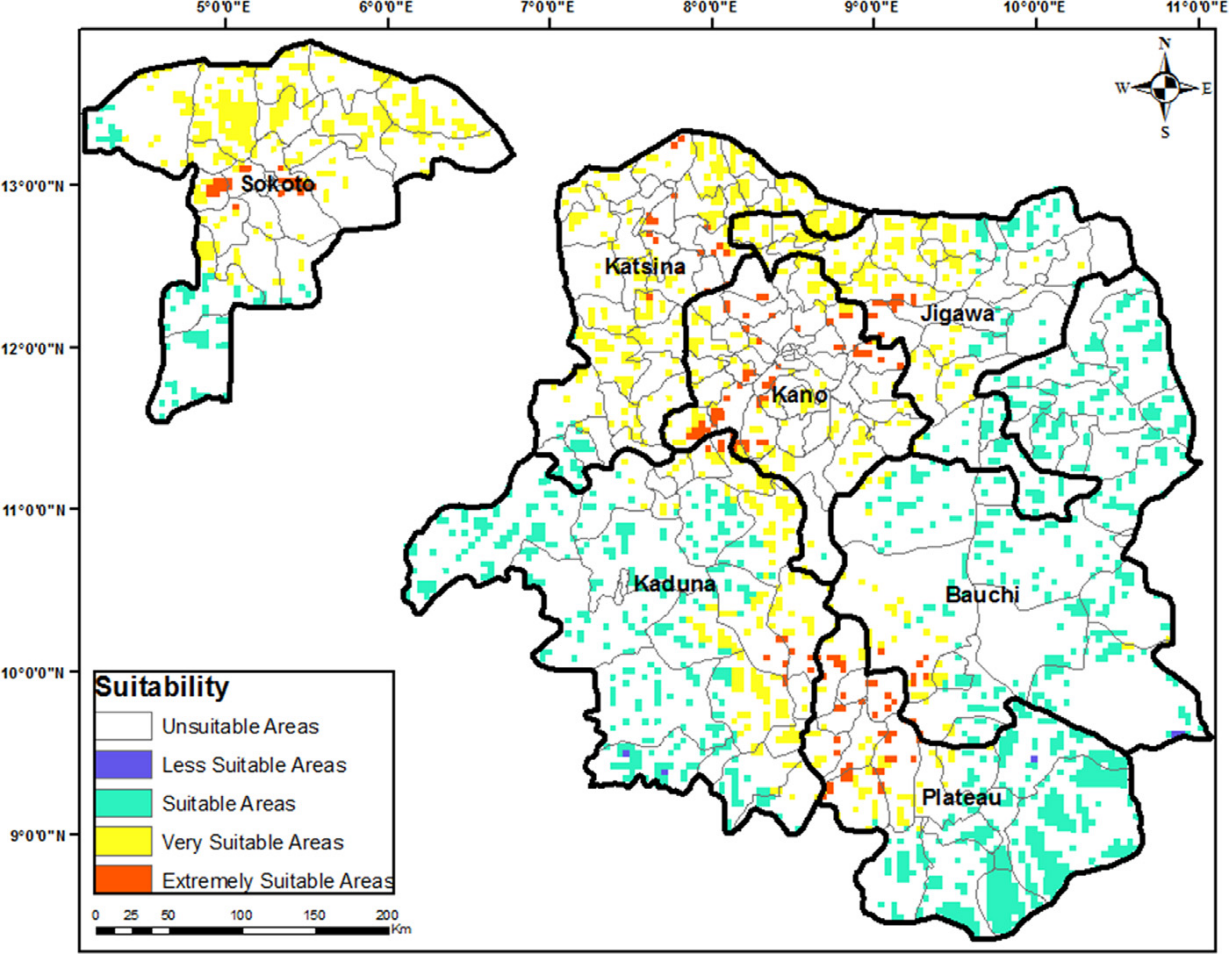
Most suitable sites for wind farm development in Northern Nigeria.

**Fig. 3:**
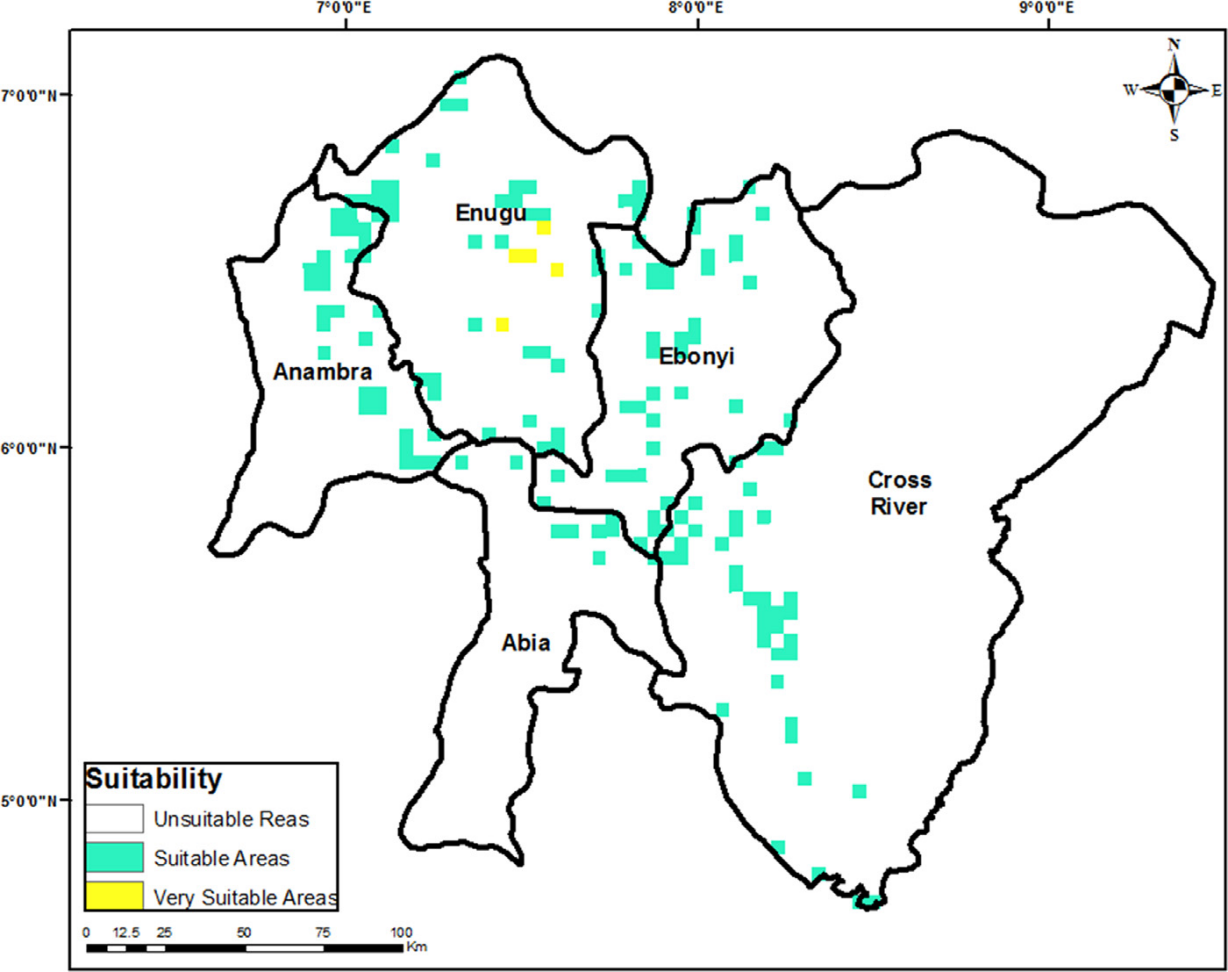
Most suitable sites for wind farm development in Southern Nigeria.

**Fig. 4:**
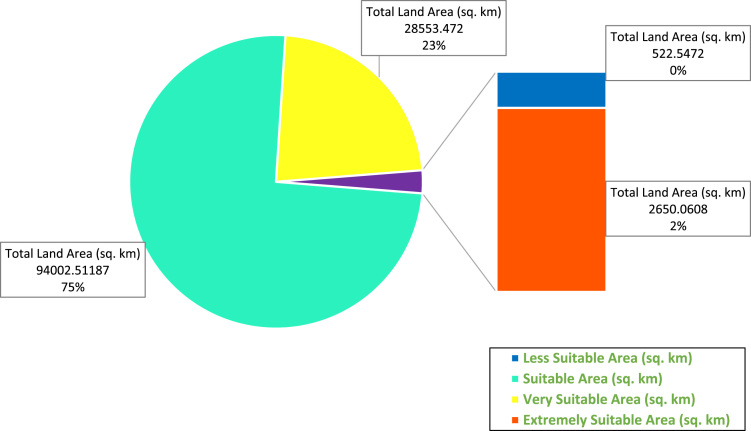
Total land area in Nigeria suitable for various wind power application.

The data contained in this paper comprises of proposed wind farm sites data obtained from wind farm site evaluation in Nigeria. The data includes wind farm suitability map for Nigeria (Fig. 1), maps of the most suitable sites for wind farm development in the northern and southern part of Nigeria (Fig. 2, Fig. 3), the total land area in Nigeria suitable for various wind power application (Fig. 4), and the total land area of the various suitability class in the most suitable states (Table 1).

**Table 1:** Total land areas of the various wind farm suitability class for the most suitable states.

**Suitability/state**	**Less suitable area (sq. km)**	**Suitable area (sq. km)**	**Very suitable area (sq. km)**	**Extremely suitable area (sq. km)**	**Total suitable areas (sq. km)**
Bauchi	0	6681.1392	765.1584	149.2992	7595.5968
Jigawa	0	1623.6288	3079.296	261.2736	4964.1984
Kaduna	37.3248	6307.8912	3079.296	242.6112	9629.7984
Kano	0	18.6624	2500.7616	1007.7696	3527.1936
Katsina	0	410.5728	4348.3392	279.936	5038.848
Plateau	18.6624	6737.1264	1101.0816	727.8336	8566.0416
Sokoto	0	1269.0432	5393.4336	429.2352	7091.712
**Sum**	**55.9872**	**23,048.064**	**20,267.3664**	**3097.9584**	**46,413.3888**

## 2 Experimental design, materials and methods

**Fig. 5:**
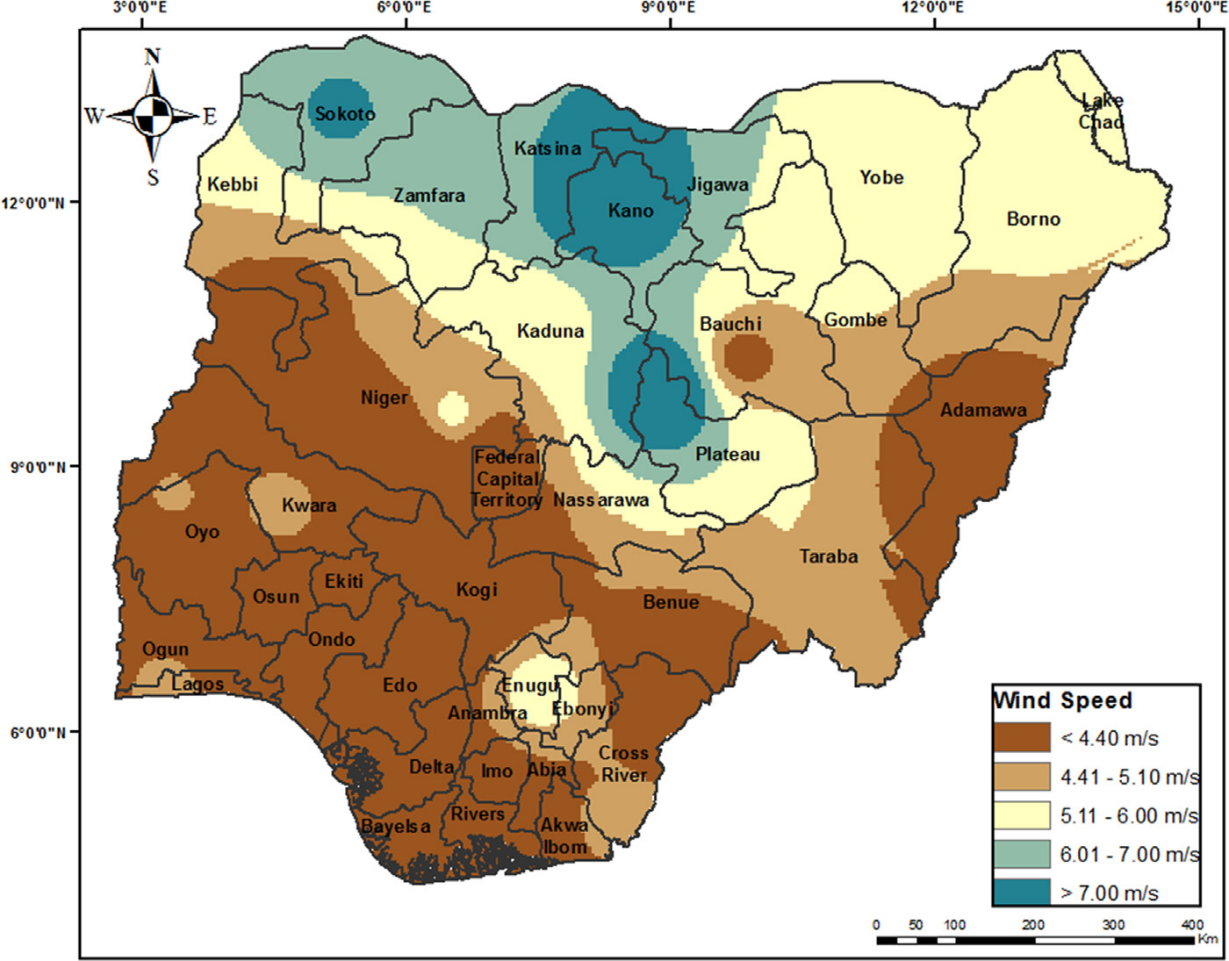
Wind speed map for Nigeria.

**Fig. 6:**
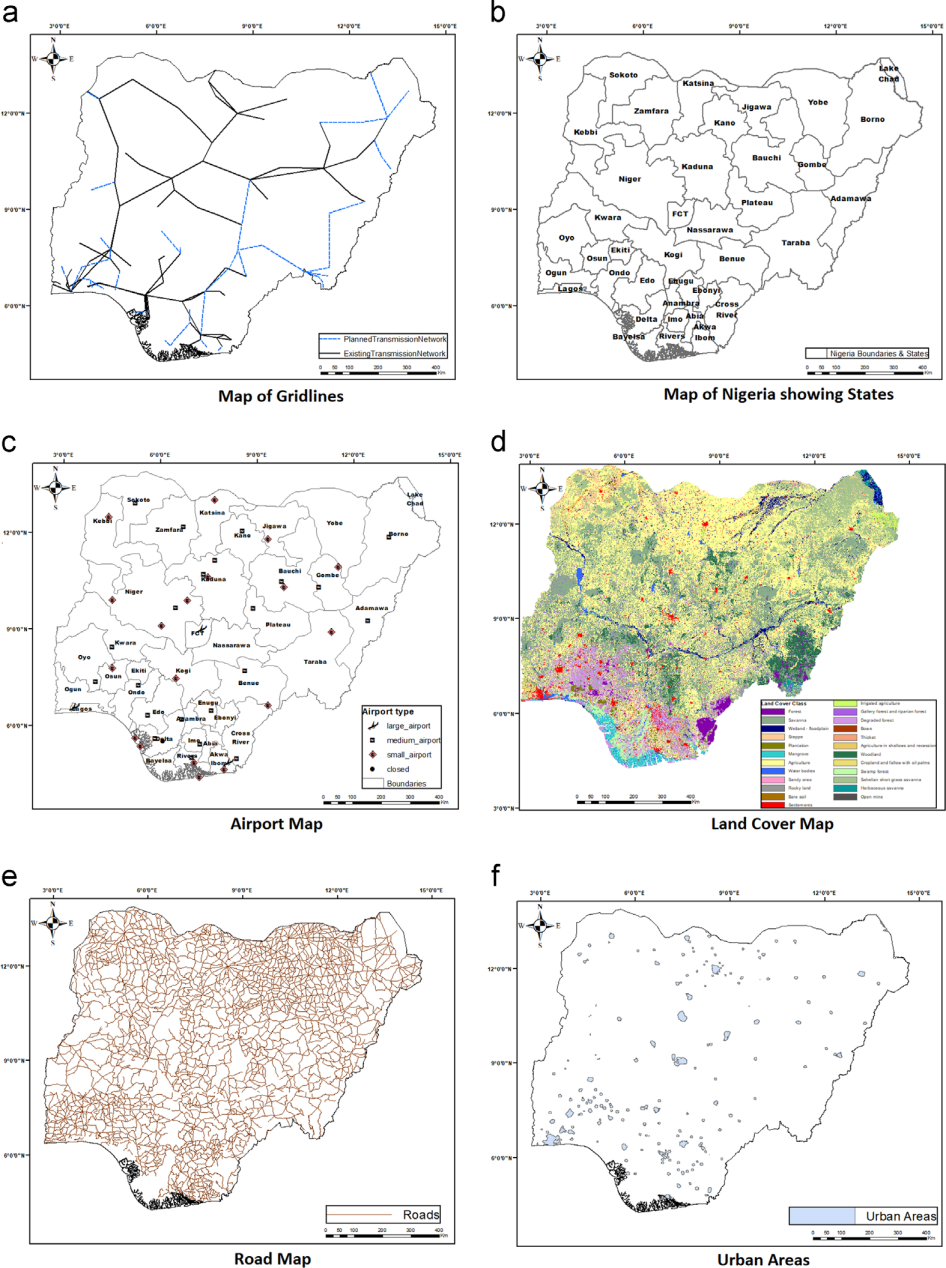

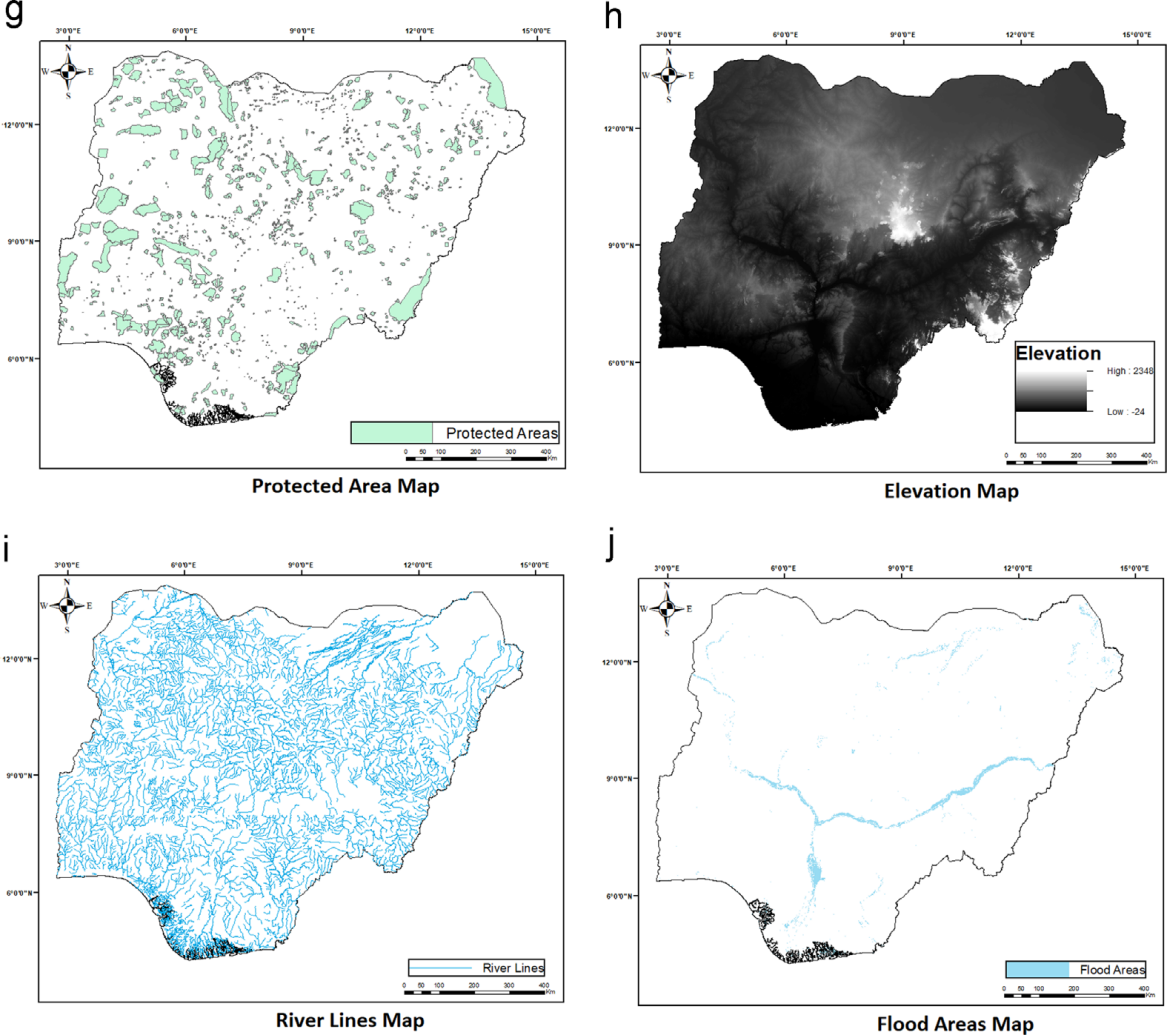
(a & b). Evaluation Data. (c & d). Evaluation Data. (e & f). Evaluation Data. (g & h). Evaluation Data. (i & j). Evaluation Data.

**Fig. 7:**
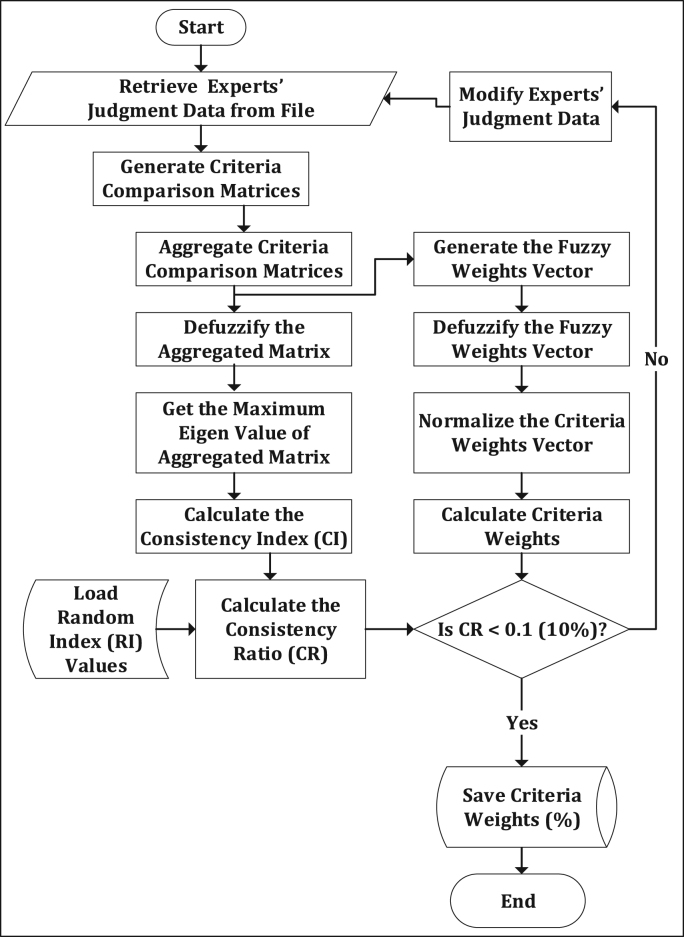
Mathematical model for generating weights of the wind farm site evaluation criteria.

The average annual wind speed data for 28 locations obtained from Nigeria Meteorological Agency (NIMET), Oshodi, Lagos, Nigeria was gotten from a previous work [1]. The data were interpolated to obtain wind speed value for other locations. The resulting map is shown in Fig. 5. The sources of other environmental and topological map data used are shown in Table 2. All evaluation data used are shown in Fig. 6(a-j) and were preprocessed using GIS software to make them usable in the wind farm site selection model. The experts’ criteria pairwise comparison (linguistic) judgments obtained from experts within and outside Nigeria are shown in Table 3. The weights of the criteria were generated using the interval type-2 fuzzy AHP (MCDM) mathematical model [2] implemented in python. The mathematical model is shown in Fig. 7.

**Table 2:** Data used and their sources.

**S/N**	**Data**	**File format**	**Source**
**1**	Wind Speed	Table	Nigeria Meteorological Agency (NIMET) as presented in a previous Research [1]
**2**	Elevation (DEM)	Raster Map	U.S Geological Survey (USGS) Earth Explorer Website
**3**	Land Cover and Flood Areas	Raster Map	Food and Agriculture Organization of the United Nations (FAO) website
**4**	Airports	Vector Map	OurAirports (https://ourairport.com/countries/NG/)
**5**	Grid Lines	Vector Map	ENERGYDATA.INFO website (An Innovation of World Bank Group)
**6**	Protected Areas	Vector Map	United Nations Environment World Conservation Monitoring Centre (UNEP-WCMC) website
**7**	Important Bird Areas (IBAs)	Raster Map	BirdLife International
**8**	Boundary, Roads, River lines, and Urban Areas	Vector Map	UN Office for the Coordination of Humanitarian Affairs (OCHA) website

**Table 3:** Experts' pairwise comparison of the weighted criteria.

**Expert's number**	**1**					**2**					**3**				
**Weighted Criteria**	**C1**	**C2**	**C3**	**C4**	**C5**	**C1**	**C2**	**C3**	**C4**	**C5**	**C1**	**C2**	**C3**	**C4**	**C5**
**Wind Speed, C1**	EE	FS	VS	AS	AS	EE	SS	VS	AS	AS	EE	FS	SS	AS	AS
**Proximity to Gridlines, C2**	1/FS	EE	FS	FS	VS	1/SS	EE	FS	SS	FS	1/FS	EE	1/SS	VS	FS
**Slope, C3**	1/VS	1/FS	EE	SS	SS	1/VS	1/FS	EE	EE	FS	1/SS	SS	EE	FS	FS
**Proximity to Towns, C4**	1/AS	1/FS	1/SS	EE	EE	1/AS	1/SS	EE	EE	SS	1/AS	1/VS	1/FS	EE	1/SS
**Proximity to Roads, C5**	1/AS	1/VS	1/SS	EE	EE	1/AS	1/FS	1/FS	1/SS	EE	1/AS	1/FS	1/FS	SS	EE
**EXPERT'S NUMBER**	**4**					**5**									
**Weighted Criteria**	**C1**	**C2**	**C3**	**C4**	**C5**	**C1**	**C2**	**C3**	**C4**	**C5**					
**Wind Speed, C1**	EE	SS	FS	AS	AS	EE	FS	FS	AS	AS					
**Proximity to Gridlines, C2**	1/SS	EE	SS	FS	VS	1/FS	EE	1/SS	SS	VS					
**Slope, C3**	1/FS	1/SS	EE	SS	FS	1/FS	SS	EE	FS	FS					
**Proximity to Towns, C4**	1/VS	1/FS	1/SS	EE	EE	1/VS	1/SS	1/FS	EE	EE					
**Proximity to Roads, C5**	1/AS	1/AS	1/FS	EE	EE	1/AS	1/AS	1/FS	EE	EE					

*EE = Exactly equal, SS = Slightly strong, FS = Fairly strong, AS = Absolutely strong, VS = Very strong, 1/SS = Reciprocal of Slightly strong, 1/FS = Reciprocal of Fairly strong, 1/AS = Reciprocal of Absolutely strong, and 1/VS = Reciprocal of Very strong.

**Fig. 8:**
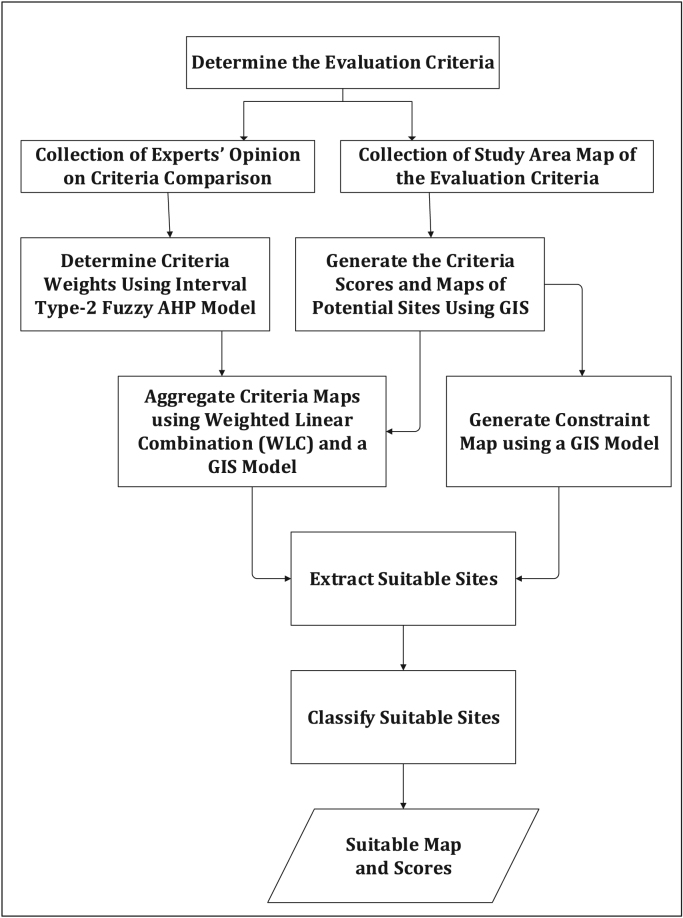
GIS-based Model for wind farm site selection.

**Fig. 9:**
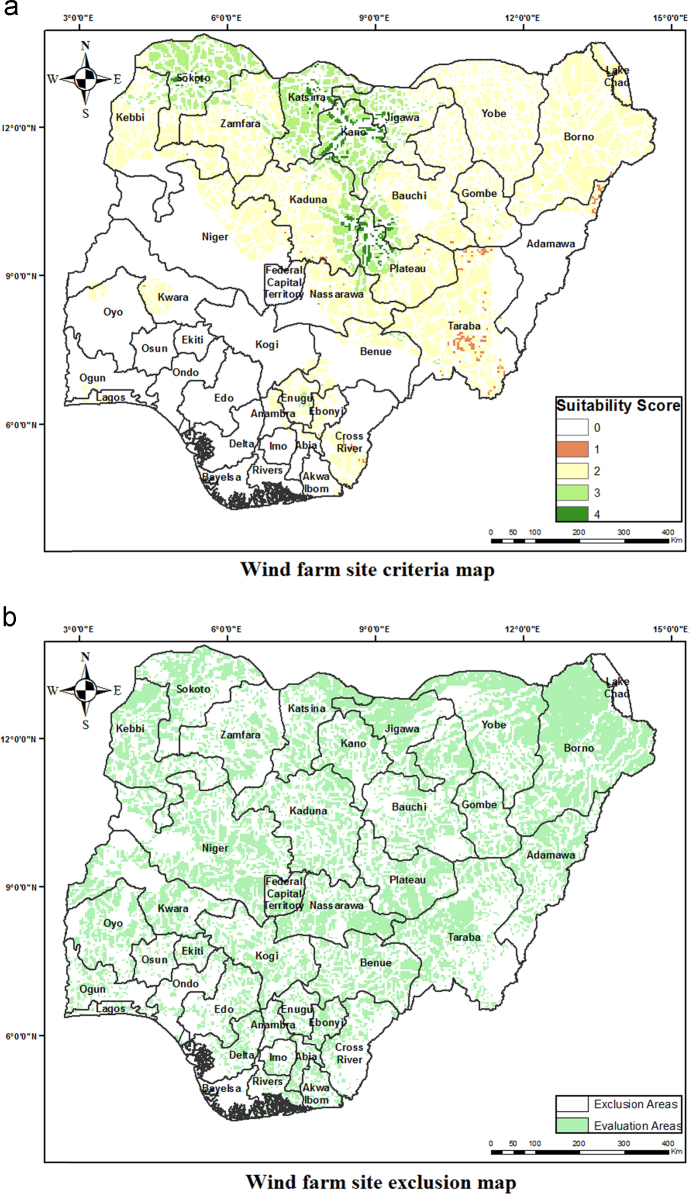
Wind farm site criteria and exclusion map for Nigeria. a. Wind farm site criteria map. b. Wind farm site exclusion map.

The criteria weights generated (Table 4), together with the preprocessed data, were inputted into the GIS-based model [3] to generate the wind farm site criteria map and exclusion map. The GIS-based model, shown in Fig. 7, is a multi-criteria model that utilizes the interval type-2 fuzzy AHP technique and it was implemented using the ArcGIS Desktop software. The weighted linear combination technique [4] was used to obtain the wind farm site criteria map by aggregating individual criteria maps. While the GIS-based model is shown in Fig. 8., the wind farm site criteria map and exclusion map are shown in Fig. 9(a & b). The classify tool in the Arc Toolbox of the ArcGIS Desktop software was used to classify the suitability map. To obtain the suitability map, the site criteria maps were overlaid with the exclusion map to exclude the exclusion areas from the wind farm site evaluation using the overlay tool [5]. To finally extract the most suitable sites from the suitability map, the extract by mask tool was used. Microsoft Excel was used to generate the pie chart for the total land areas of the suitability classes. The criteria used in defining the suitability maps presented in Fig. 1, Fig. 2, Fig. 3 are shown in Table 5.

**Table 4:** Wind farm site evaluation criteria weights.

**Criteria**	**Symbol**	**Calculated weight**
Wind Speed	C_1_	0.4974
Proximity to Gridlines	C_2_	0.2449
Slope	C_3_	0.1681
Proximity to Towns	C_4_	0.0519

**Table 5:** Criteria for suitability maps in Fig. 1, Fig. 2, Fig. 3.

**Proximity (m) to**			**Wind Speed (m/s)**	**Slope (%)**	**Elevation (metres)**	**Score**	**Suitability**
**Roads**	**Gridlines**	**Towns**					
< 500	< 250	< 2,000	< 4.4	> 15.0	2000–2384	0	Not Suitable
15,000.1–20,000	> 20,000	2,001–6,000	4.4–5.1	10.1–15.0	1000–2000	1	Less Suitable
10,000.1–15,000	10,001–20,000	6,001–10,000	5.1–6.0	6.1–10.0	500–1000	2	Suitable
5,000.1–10,000	5001–10,000	10,001–20,000	6.0–7.0	3.1–6.0	200–500	3	Very Suitable
500.1–5,000	251–5000	> 20,000	> 7.0	< 3.0	> 200	4	Extremely Suitable
**Airports**	**Important Bird Areas**	**Protected Areas**	**River Lines**	**Land Cover**		**Score**	**Classification**
Within 5000 m buffer	Within 300 m buffer	Within 500 m buffer	Within 200 m buffer	Forests, woodlands, and wetlands		0	Excluded Areas
Otherwise	Otherwise	Otherwise	Otherwise	Otherwise		1	Evaluation Areas
