# Supplementary material for: On the most suitable sites for wind farm development in Nigeria
Source: Data Brief. 2018 May 8;19:29–41. doi: 10.1016/j.dib.2018.04.144 (PMC5992976; doi:10.1016/j.dib.2018.04.144)
Supplement: Supplementary file 1 — Transparency document [file mmc1.docx]

**Conflict of Interest**

The authors declare that there is no conflict of interest concerning the publication of this manuscript
